# An analysis of pediatric retinoblastoma medical malpractice litigation

**DOI:** 10.1371/journal.pone.0342114

**Published:** 2026-06-04

**Authors:** Victoria Miller, Alyssa D. Reese, Katherine Foote, Alejandro Navarro, Andrew Galfano, Christian Hietanen

**Affiliations:** 1 Touro College of Osteopathic Medicine, Middletown, New York, United States of America; 2 Division of Plastic and Cosmetic Surgery, Department of Surgery, Macon & Joan Brock Virginia Health Sciences at Old Dominion University, Norfolk, Virginia, United States of America; 3 Jacobs School of Medicine and Biomedical Sciences, University at Buffalo, Buffalo, New York, United States of America; University of Health and Allied Sciences, GHANA

## Abstract

**Background:**

Retinoblastoma is a cancer of the retina that is most commonly diagnosed in the pediatric population. Unfortunately, this cancer can spread, leading to poor outcomes such as enucleation. The purpose of this study was to determine the characteristics of medical malpractice litigation associated with a diagnosis of retinoblastoma in the pediatric population.

**Methods:**

A search for all available cases associated with “retinoblastoma” in the Westlaw Campus Research Legal Database was conducted. This spanned from database inception to August 2023. Cases were included if there was a complaint of medical malpractice related to retinoblastoma present in a pediatric patient. Demographic information for each patient was collected. The specialty of the defendant(s), state where the case took place, and case outcomes were also determined.

**Results:**

Thirteen cases with a total of 14 patients were analyzed. One case included two siblings who were both diagnosed with retinoblastoma. The majority of the patients were female (N = 10, 71.4%) and all patients were alive at the time of trial. Three of the cases were initiated because an obstetrician-gynecologist/reproductive endocrinologist or ophthalmologist failed to tell a parent about the genetic risks associated with a family history of retinoblastoma. Specialties of the defendant(s) most frequently involved pediatrics (N = 5, 38.4%) and family medicine (N = 4, 30.8%).

**Conclusion:**

Failure to diagnose retinoblastoma represents the most common context in which malpractice litigation arises in pediatric patients. However, few cases were identified in this study, which may be due to the settlement of these cases out of court. Although retinoblastoma is a condition primarily diagnosed in the pediatric population, our analysis shows that many different specialties are potentially involved during litigation. This highlights the importance of retinoblastoma education and screening across multiple specialties.

## Introduction

Retinoblastoma is a rare and malignant pediatric cancer that typically presents with initial symptoms of leukocoria and strabismus [1]. In most developed countries, the survival rate of children diagnosed with retinoblastoma is greater than 95% [2]. However, when left untreated, this cancer can be fatal [3,4]. At end stages, tumors will grow into the skull, affecting the innermost regions of the eye and optic nerve. Thus, early diagnosis and proper treatment are crucial to preserve vision and improve the likelihood of survival.

Despite advancements in the care of children with retinoblastoma, challenges surrounding timely and accurate diagnosis of this condition continue to exist. For example, unusual presentations, including vitreous hemorrhage and inflammation, can mask the condition and lead to delayed intervention. These challenges highlight the need for additional insight into potential areas for improvement in the diagnostic process, with a focus on patterns associated with suboptimal outcomes.

One method for identifying these patterns is through the evaluation of medical malpractice litigation. One previous study of malpractice litigation in ocular oncology identified two cases associated with retinoblastoma [5]. The purpose of this study was to provide an updated analysis of the characteristics of medical malpractice litigation associated with a diagnosis of retinoblastoma in the pediatric population.

## Materials and methods

### Study design and case selection

A search for all available state and federal cases associated with “retinoblastoma” in the Westlaw Campus Research Legal Database (Thompson Reuters, New York, NY) was conducted in August 2023. The Westlaw Campus Research Legal Database includes jury verdict reports as well as summary statements for each case. However, the database does not include cases that were settled outside of court or dropped prior to court. Due to licensing restrictions imposed by the database provider, full case texts cannot be shared publicly. The search results and included case data for this study can be found in Supporting Information (Supporting Information 1 and 2). This research was classified as non-human research that did not require approval per institutional review board guidelines.

The following search terms were utilized: (“retinoblastoma”) & (“malpractice” OR “negligence”). No limitation was placed on year of the case documents; cases from database inception to August 2023 were included in the search. Cases were included if there was a complaint of medical malpractice related to retinoblastoma present in a pediatric patient with an age of less than 18 years. Cases were excluded if limited information was available, the patient was an adult, or the complaint did not involve medical malpractice related to retinoblastoma.

### Data collection

Demographic data for each patient were collected, including age, sex, and vital status (alive or deceased) when available. The survey collected the following information for each case: case title, state of case, year, plaintiff relationship to patient, gender, patient’s life status and age, result of the verdict, result of the appeal, description of outcomes, specialty involved, type of negligence, resulting injury, description of case, and amount rewarded. Not all descriptive features in the survey were included in the final study if there was a lack of information in a majority of the articles.

### Data analysis

Data was screened and collected and independently verified by two authors (AR and VM). All articles resulting from the initial search were screened with the inclusion/exclusion criteria previously stated. For cases that were ultimately included, data was extracted utilizing a standardized Google Form and exported to Google Sheets. Descriptive statistics were calculated using a combination of Google Sheets and manual calculations, consisting of summation of results and percentages, and reviewed by two authors (VM and AR).

## Results

The database search yielded 50 cases, with 47 cases for review after removal of duplicates. Ultimately, 13 cases with 14 patients were reviewed that met inclusion criteria; one case included two siblings who were both diagnosed with retinoblastoma. Case documents were dated from 1978 to 2020. Cases were most frequently initiated in New York (N = 2, 14.2%), New Jersey (N = 2, 14.2%), and Pennsylvania (N = 2, 14.2%). Additional locations included California (N = 1, 7.1%), Connecticut (N = 1, 7.1%), Illinois (N = 1, 7.1%), North Carolina (N = 1, 7.1%), Rhode Island (N = 1, 7.1%), Texas (N = 1, 7.1%), and Washington (N = 1, 7.1%).

The patient’s parents were the plaintiffs in all the cases. The patient population had an average age of 1.3 years (SD: 1.3 years) and consisted mainly of females (N = 10, 71.4%) (Table 1). All patients in this study were alive at the time of trial and no deaths were reported. Additional patient demographics can be found in Table 1.

**Table 1:** Patient Demographics.

Demographics		N (%)
Gender	Male	3 (21.4)
	Female	10 (71.4)
	Unknown	1 (7.1)
Age (years)	0-1	6 (42.9)
	2-3	1 (7.1)
	4-5	1 (7.1)
	Unknown/ Unborn	6 (42.9)

Failure to diagnose pediatric retinoblastoma accounted for 10 (76.9%) of the malpractice claims. Three (23.1%) of the cases were initiated because an obstetrician-gynecologist or ophthalmologist failed to tell a parent about the genetic risks associated with a family history of retinoblastoma. Specialties of the defendants included optometry (N = 1,7.7%), ophthalmology (N = 2, 15.4%), pediatrics (N = 5, 38.5%), family medicine (N = 4, 30.8%), obstetrics-gynecology (N = 2, 15.4%), pathology (N = 1, 7.7%), and reproductive endocrinology (N = 1, 7.7%) (Table 2). However, two cases were ruled in favor of the plaintiff based on failure to diagnose retinoblastoma and delayed diagnoses; enucleation occurred in one case and repeated radiation was performed in the other.

**Table 2:** Case and Patient Characteristics.

*Case #*	*Patient #*	*State*	*Year*	*Sex*	*Age*	*Specialty Involvement*	*Malpractice Claim*	*Result of Appeal*
***Initial Verdict: For Defendant***								
A	1	NJ	1988	Female	4 y	Optometrist	Informed Consent, Failure to diagnose	Appealed and remanded
B	2	NY	1998	Female	NA	Ophthalmologist	Informed Consent – Failure to inform patient’s parent(s) about genetic risks associated with retinoblastoma	NA
	3	NY	1998	Female	NA			
C	4	TX	2020	Male	1 y	Pediatrics	Failure to diagnose	Appealed and affirmed
D	5	NC	1988	Female	6 m	Family Medicine	Failure to diagnose	NA
E	6	IL	2003	Female	4 m	Pediatrics	Failure to diagnose	Appealed and affirmed
F	7	PA	1987	Male	NA	Pediatrics	Failure to diagnose	Appealed and affirmed
G	8	CT	2012	Male	9 m	Pediatrics	Failure to diagnose	Appealed and affirmed
H	10	NJ	1990	Female	NA	OBGYN	Informed Consent – Failure to inform patient’s parent(s) about genetic risks associated with retinoblastoma	Appealed and remanded
I	12	CA	2020	Female	6 m	Reproductive endocrinology	Informed Consent – Failure to inform patient’s parent(s) about genetic risks associated with retinoblastoma	Appealed and remanded
Initial Verdict: For Plaintiff								
J	11	WA	2015	Unknown	NA	Family Medicine	Failure to diagnose	NA
Initial Verdict: Unknown								
K	9	NY	2009	Female	6 m	Family Medicine	Failure to diagnose	NA
L	13	RI	2003	Female	3 y	Ophthalmologist, Family Medicine, Pathology	Failure to diagnose	NA
M	14	PA	1996	Female	NA	Pediatrics	Failure to diagnose	NA

## Discussion

The screening and detection of pediatric retinoblastoma often occur in the setting of a visit with the child’s pediatrician, as this rare cancer is often identified before a patient is two years old [6]. Nevertheless, there are instances where diagnosis may prove challenging, leading to medical consequences and subsequent claims of malpractice. This study analyzed malpractice cases surrounding pediatric retinoblastoma to identify trends in claims and risk factors relevant to clinical practice.

Malpractice cases are based upon a standard legal framework with some variation between states. In general, to initiate a claim of malpractice, a plaintiff must show that there was (1) a duty of care established through a physician-patient relationship; (2) a breach of the standard of care, typically defined as what a reasonable clinician would do under similar circumstances; (3) causation, meaning that the physician’s breach of their duty directly harmed the patient; and (4) damages, such as increased treatment burden. These elements are broadly applied across jurisdictions.

Failure to diagnose was a common allegation of malpractice lawsuits concerning pediatric retinoblastoma in the present study. This corroborates the work of Wallace *et al.* who investigated the epidemiology of malpractice claims in primary care specialties in 2013 [7]. In their study, cancer was among the most frequently reported missed diagnoses in both children and adults that prompted litigation [7]. The failure to diagnose claims in pediatric patients were most frequently associated with the fields of pediatrics and family medicine in both our study and Wallace et al.’s study as well. This is likely because these are the sole medical specialties that the average child will visit during their early life.

When retinoblastoma is treated appropriately, there is a chance that the patient’s vision can be saved in either one or both eyes that are affected; 99% of children retain at least one eye and more than 90% retain normal vision in the affected eye [8]. Therefore, preventing a missed or delayed diagnosis is crucial to improving the outcome of children diagnosed with retinoblastoma. To optimize this, electronic medical record systems could potentially incorporate a best-practice alert that prompts the clinician to review family history for retinoblastoma or conduct additional testing to ensure due diligence.

Improper diagnosis can occur when a provider lacks knowledge or has poor judgment, and can be related to failure to obtain a detailed history, perform the necessary physical examination, order the appropriate diagnostic test, create an adequate follow-up plan, or communicate information to medical staff and the patient [7]. A quality analysis of medical errors in family practice found that 13.4% of physicians reported that errors were due to gaps in knowledge or skills, while 82.6% were due to healthcare system dysfunction [9]. Thus, improvement in clinical skills, knowledge, communication, and collaboration between providers can likely decrease failure to diagnose. Multiple studies have identified the need for transparency to improve the patient-provider relationship and prevent the filing of medical malpractice claims [10]. Primary care physicians who expressed more empathy, attempted to connect with their patients, and spent more time during appointments were less likely to have had a malpractice claim [10]. This again emphasizes the importance of personalization and collaboration with patients in their care to prevent feelings of neglect and uncertainty.

Failure to diagnose and improper diagnosis may establish the breach of standard of care if the plaintiff, the patient or their family member, can show that a reasonable, knowledgeable clinician should have identified early signs of the condition. Courts, including judges and juries, frequently rely on clinical guidelines and expert testimony to establish the standard of care and demonstrate that a physician has failed to uphold it. In jury trials, jurors may weigh the credibility of the experts provided by the plaintiff and defendant as well as the severity of the patient’s harm. In contrast, judges often focus on the four elements of malpractice and evaluate whether there is sufficient evidence to support each component.

Proper screening protocols are a crucial component of upholding standards of care surrounding pediatric retinoblastoma. The red reflex test is a common screening for newborns for ocular pathology, including retinoblastoma [11]. The test is a non-invasive ocular examination that involves observing the child’s pupils with an ophthalmoscope in a dark room [12]. Any asymmetry noted is considered abnormal and requires further investigation [12]. However, it is in general agreement among physicians that testing the red reflex has poor sensitivity in diagnosing malignancies of the eye. In a study conducted by Subhi *et al.*, 25.3% of infant patients with an ocular disease, including retinoblastoma, had a normal red reflex [11].

There are several reasons why a red reflex test may result in inconsistent findings. Smaller pupils that do not allow as much light to pass, decreased light entering the eye, and diseases of the peripheral retina can all result in inaccurate findings during the red reflex test in pediatric patients [11]. Additionally, adequate training is needed to perform the exam and each examiner possesses a different level of skill set [12,13]. This skill set impacts not only diagnosis but also appropriate ophthalmic referral and timely treatment.

Despite the low sensitivity of the red reflex test, it is crucial that pediatricians and family medicine physicians perform this test during a routine, pediatric physical exam. Although a normal red reflex will not always indicate the absence of disease, there is a strong likelihood that an abnormal red reflex is indicative of an ocular disease. In one case within the present study, the plaintiff claimed that the defendant, a pediatrician, was negligent because they did not perform the red reflex test. The outcome of this trial was not publicly available, suggesting that the case was settled out of court.

As an alternative to conducting the red reflex test, screening for retinoblastoma can be accomplished with wide-field digital retinal imaging (WFDRI). WFDRI allows for imaging of the retina and can detect peripheral lesions, unlike the red reflex test [14]. While WFDRI may offset some of the limitations of the red reflex test, it does not possess the ease of implementation, low cost, and availability to primary care providers.

A handful of cases in the present study arose due to claims of failure to provide informed consent related to genetic risks associated with retinoblastoma. Retinoblastoma has a hereditary component and genetic testing can be utilized to predict the likelihood that a couple’s offspring will be diagnosed with retinoblastoma. Overall, in the cases of failure to warn the patient of genetic risks, all of the cases were ruled in favor of the defendant. Nevertheless, vigilance on behalf of obstetrics/gynecologists, pediatricians, and primary care providers to acknowledge genetic risks of retinoblastoma and proceed accordingly with family planning is necessary. This includes adoptive or donor-conceived families who may not know their familial genetic history and be unaware of heritable conditions.

Beyond pediatric retinoblastoma, these cases underscore the malpractice risk associated with failure-to-diagnose claims across several specialties. The complications that delay and impede a timely diagnosis may arise when a patient presents long after symptoms first appear or, conversely, far too early, when available diagnostic tools may not yet be able to detect the condition [15]. Because diseases can present differently in each individual, there are additional opportunities for missed or delayed diagnosis [15]. These factors are relevant to this study’s findings, as several cases involved failure to perform a red reflex test, inadequate recognition of risk factors, or failure to refer to an ophthalmologist.

One case in this study was ruled in favor of the plaintiff and involved an optometrist as the defendant. This individual was the only optometrist noted in the 13 cases. The defendant allegedly did not refer the plaintiff to an ophthalmologist promptly when the pediatric patient developed a right-eye white reflex. The patient was later diagnosed with retinoblastoma and underwent enucleation. Since, the presence of a white reflex mandates an urgent referral to ophthalmology, in this instance, the provider failed to meet the applicable standard of care [16].

This case highlights the importance of referrals to specialists, specifically in instances where the requirements of care are outside the scope of the provider. There are approximately double the number of optometrists to ophthalmologists in the United States [17]. Thus, it is crucial to differentiate the roles of each eye care provider and distinguish when a patient requires more advanced medical attention, as is provided by an ophthalmologist. This can benefit the care of children with undiagnosed retinoblastomas to preserve their eyesight and reduce mortality.

### Limitations

The study was limited by the inherent issues related to the nature of the Westlaw Campus Research legal database. This may introduce selection bias and generalizations, as this database is not all inclusive and is limited to cases with verdict reports. It does not include cases that were settled out of court or dropped, and is often missing documentation related to the cases. Therefore, cases of gross negligence on behalf of the provider may have been settled out of court. Since this study was conducted nationwide, the legal criteria and jurisdiction surrounding medical malpractice litigation may also vary state to state.

## Conclusion

Failure to diagnose retinoblastoma represents the most common context in which malpractice litigation arises in pediatric patients. Few cases were identified in this study, which may be due to the settlement of these cases out of court. Although retinoblastoma is a condition primarily diagnosed in the pediatric population, many different specialties can potentially be involved in malpractice litigation. This underscores the importance of retinoblastoma education, screening, and genetic testing across multiple specialties.

## Supporting information

S1 File Comprehensive list of unique retinoblastoma malpractice cases identified during the legal database search process. (CSV)

S2 File Dataset of retinoblastoma-related malpractice cases included in the study, including jurisdiction, year, patient demographics, malpractice allegations, specialty involved, verdict outcomes, and associated case characteristics. (CSV)
